# Artery of Percheron Infarction as an Unusual Cause of Korsakoff's Syndrome

**DOI:** 10.1155/2015/927809

**Published:** 2015-11-24

**Authors:** Yongxing Zhou, Derrick Fox, Abhishek Anand, Amal Elhaj, Arushi Kapoor, Faranak Najibi, Han Kim, Roger Weir, Annapurni Jayam-Trouth

**Affiliations:** ^1^Department of Neurology, Howard University Hospital, 2041 Georgia Avenue NW, Washington, DC 20060, USA; ^2^Department of Psychiatry, Howard University Hospital, 2041 Georgia Avenue NW, Washington, DC 20060, USA; ^3^Department of Radiology, Howard University Hospital, 2041 Georgia Avenue NW, Washington, DC 20060, USA

## Abstract

The Korsakoff syndrome is defined as “an abnormal mental state in which memory and learning are affected out of all proportion to other cognitive functions in an otherwise alert and responsive patient.” Confabulation refers to false or erroneous memories arising, not deliberately, in the context of a neurological amnesia and is often thought of as pathognomonic of the Korsakoff syndrome. Although the exact pathophysiology is unknown, various studies have identified brain lesions in the thalami, mammillary bodies, and frontal cortex. We report a case of a 68-year-old male presenting with acute altered mental status on July 16, 2015. The neuropsychological dysfunctions included prominent Korsakoff's syndrome, which became apparent when the altered mental status resolved. Amnesia was accompanied by prominent confabulation, disorientation, and lack of insight into his own disability. Neuroradiological data indicated that the intralaminar and dorsomedial nuclei in bilateral thalami were infarcted by occlusion of the artery of Percheron. We believe that ours is one of few reported cases of Korsakoff syndrome in a patient with infarction involving the territory of the artery of Percheron. We conclude that bilateral thalamic lesions could cause Korsakoff's syndrome and the intralaminar and dorsomedial nuclei might be important structures in the pathogenesis of confabulation.

## 1. Introduction

**Figure 1:**
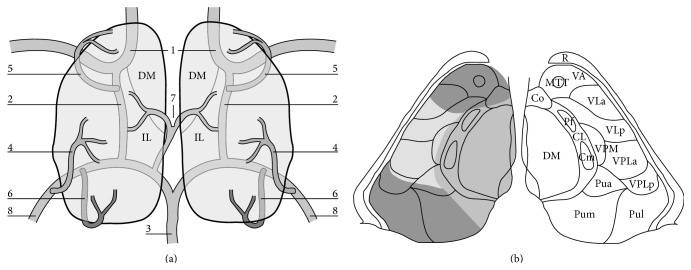
Schematic view of the arterial supply to the thalamus (adapted with permission from [6]). (a) The view from above; (b) detailed relationship between the arterial territories and nuclear subgroups within the thalamus. 1: carotid artery; 2: posterior communicating artery; 3: basilar artery; 4: thalamogeniculate arteries; 5: tuberothalamic artery; 6: posterior choroidal artery; 7: paramedian pedicle (AOP); 8: posterior cerebral artery. Main thalamic nuclei and tracts: CL: central lateral; CM: centromedian; Co: commissural; Cp: commissural posterior; DM: dorsomedian; MTT: mammillothalamic tract; Pua: pulvinar anterior; Pum: pulvinar medial; Pul: pulvinar lateral; Pf: parafascicularis; R: reticular; VA: ventral anterior; VLa: ventral lateral anterior; VLp: ventral lateral posterior; VPLa: ventroposterolateral anterior; VPLp: ventroposterolateral posterior; VPM: ventroposteromedian.

Percheron described four major vascular territories of the thalamus [1]. These are the (1) tuberothalamic, (2) inferolateral, (3) paramedian, and (4) posterior choroidal vessels (Figure 1). The artery of Percheron (AOP) arises from a common trunk of one P1 segment of the posterior cerebral artery (PCA) and supplies the thalamus bilaterally [2–5] (Figure 1, number 7). Occlusion of the common trunk leads to typical mirror-like symmetric bilateral thalamic infarctions which can be considered pathognomonic of occlusion of the artery of Percheron. The classic features of acute paramedian infarctions include altered mental status, vertical gaze paresis, and cognitive impairment [3, 6]. The neuropsychological dysfunctions become apparent when the decreased level of consciousness resolves. Often there is no focal neurological deficit, making this diagnosis easily missed in the acute setting. Here, we report a case of bilateral paramedian thalamic infarctions presenting acutely to our Emergency Department (ED) with acute confusion state, neuropsychological dysfunction including amnesia, and accompanying confabulation which was evident after the patient regained consciousness and discuss the clinical presentation due to AOP infarction as well as diagnostic and management challenges.

## 2. Case Report

**Figure 2:**
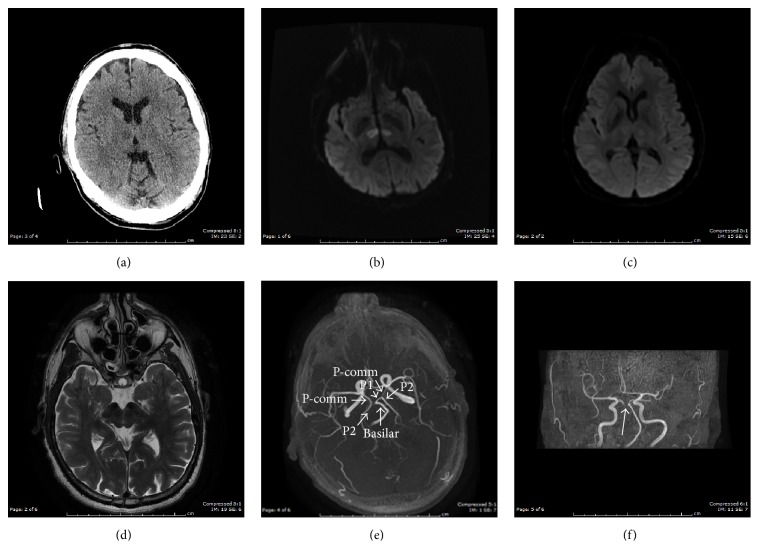
(a) CT head on July 16, 2015, in ER showed bilateral thalamic hypodensities which may represent infarcts; (b) MRI (DWI) on July 16, 2015, showed acute bilateral medial thalamic infarcts, consistent with artery of Percheron territory; (c) MRI (DWI) after two weeks of MICU management on July 31, 2015, showed improved; (d) T2-weighted MRI showed no midbrain involved; (e, f) MRA showed occluded or atretic right P1 segment. The right PCA is patent and supplied by the P-comm. The left PCA is patent.

**Figure 3:**
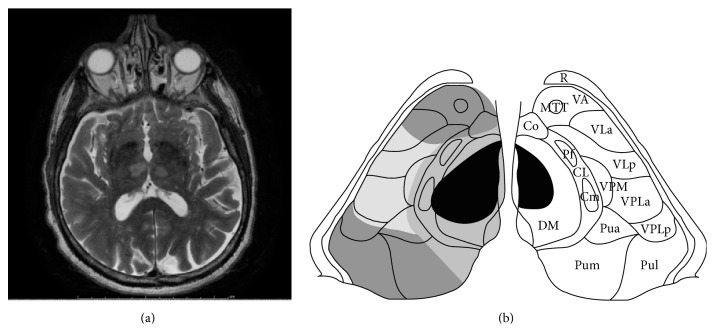
(a) T2-weighted MRI showed a bilateral paramedian; it was done the same day when patient became comatose on July 16, 2015. Patient has amnesia accompanying confabulation after the altered mental status resolved. (b) Schematic representation of the lesion in bilateral thalamic DM and IL nuclei (see Figure 1 for legend for detail).

A 68-year-old right handed male (Mr. C. R.) with past medical history of hypertension and chronic back pain was brought to the ED via EMS unresponsive after being found on the street at 6:30 a.m. on July 16, 2015. In the ED he was afebrile, with a blood pressure of 172/110 mmHg, heart rate of 87 beats/min, and respiratory rate of 17 breaths/min. He was drowsy and arousable, but not cooperative for a complete physical examination. The pupils were pinpoint, 1 mm, and reactive to light. He moved his limbs in response to painful stimuli. There was no focal neurological deficit except for probable vertical gaze paresis. Laboratory tests, including blood glucose, full blood count, electrolytes, liver, and renal function tests, were unremarkable. Electrocardiogram (EKG) showed a normal sinus rhythm. CT head showed a faint hypodense lesion in both thalami (Figure 2(a)). He was admitted to the medical intensive care unit (MICU). Diffusion-weighted magnetic resonance imaging (MRI) performed on the same day demonstrated hyperintensities bilaterally in the thalamus, consistent with restricted diffusion secondary to an acute ischemic stroke in the artery of Percheron (AOP) territory (Figures 2(b) and 3(a)). MR angiography demonstrated occluded or atretic right P1 segment of the PCA. The right PCA was patent and supplied by the posterior communicating artery (P-comm). The left PCA was patent (Figures 2(e) and 2(f)). He was initially comatose in the MICU but gradually regained consciousness and was transferred to the floor after 12 days. The EEG at transfer showed a mildly abnormal awake and drowsy EEG record with diffuse slowing and no paroxysmal features.

During his hospitalization, the patient developed atrial fibrillation and difficult to control hypertension. He had behavior changes with agitation and aggression, as well as cognitive and communication impairment. Detailed neurological examinations over several days showed fluctuating arousal and orientation as well as impaired learning and memory including impaired anterograde and retrograde memory. At some point he had MMSE score of 15/30. Language was impaired with problems in multiple areas including paraphasia and monotonous speech with hypophonia. He had no limb dyspraxia bilaterally for scissors, screwdriver, and hammer. Facial praxis was normal without any abnormalities for drinking through a straw or blowing out a match. Visuospatial examination was normal for clock, notebook, television, and wall calendar and he had no neglect. His affect was flat and apathetic at times and happy at other times. He initially demonstrated auditory and visual hallucinations, repeatedly pointing to nonexistent “shoes” to be brought over so he could get up and visit his wife; he saw “smoke” outside the window and heard unidentifiable voices. He saw people outside his room walking backwards and Prophet Mohamed speaking to him. He described the characters he saw and assumed that they spoke to him. His cranial nerves were normal except for vertical gaze paralysis. His motor function, reflexes, extremity coordination, and sensory system were all normal. Gait could not be assessed as the patient needed restraints.

During his hospital course he demonstrated confabulation, repeatedly talking about the need to go home and rest so he could carry on with his nonexistent “job” as a delivery man that he had to feed his wife because his son had passed away and no one else was working at home so he was responsible; however, sometimes he said that he was married and on other days he was not. He claimed his wife's name was Lisa and she lived in DC once but failed to answer why she was not visiting him. He might have had a son who died or might not, but he has never married, as confirmed by his brother. He stated he was a building kitchen supervisor with three women working for him, whom he had to chastise for bad behavior. On another evaluation he insisted that he was a Home Health Aide and he took care of patients in the Home Health Aide facility of this hospital that he had been doing this job for 1 year and he worked from 10 a.m. to 2 p.m. He also showed fluctuating orientation to month, year, and his whereabouts. He had fluctuating speech varying from mumbling to highly descriptive speech, using complex sentences and appropriate words. His attention and focus varied as did his recent recall and ability to retain in memory recent events. His visual and auditory hallucinations responded to treatment with olanzapine; however the confabulations persisted throughout his hospital stay.

## 3. Discussion

Bilateral paramedian thalamic infarction due to AOP occlusion is a rare cause of altered mental status. Our case indicates the importance of considering AOP territory ischemic stroke in the differential diagnosis of acute decreased level of consciousness in the elderly. In the acute setting, the symptoms are very similar to nonconvulsive status epilepticus, metabolic/toxic encephalopathy, and encephalitis. A careful interview of the family for the course of the consciousness impairment will help in the diagnosis. Our case presented to the ED with unresponsiveness of unknown onset, with no family members available in the ED. His physical examination showed no focal neurological deficit. He had pinpoint pupils making the diagnosis mimic toxic encephalopathy. Other differential diagnoses considered in our case were “top of the basilar artery” syndrome, deep cerebral venous thrombosis (DCVT), hypertensive encephalopathy, Wernicke's encephalopathy, extrapontine myelinolysis, West Nile/Japanese encephalitis, and Creutzfeldt-Jakob disease [7]. However, they were excluded based on the clinical presentation, physical examination, and imaging studies.

The paramedian artery (AOP) ascends within thalamus from its medial and ventral aspect to its lateral and dorsal part. Infarcts in the paramedian territory involve mainly the dorsomedian and intralaminar nuclei (Figures 1(a) and 1(b)). Three classic clinical features are seen: altered mental status, cognitive impairment, and vertical gaze paresis, which were all observed in our case throughout his fluctuating clinical course [6]. A variety of clinical signs including dysarthria, oculomotor disturbance, impaired convergence, retraction of eyelids, hemiplegia, ataxia, and involuntary movement of limbs have been described [8]. The neuropsychological dysfunction becomes apparent when the altered mental status resolves. These patients mainly present with personality changes, disinhibited behavior associated with apathy, loss of self-activation, and amnesia. An analysis of 120 cases showed major neuropsychological findings including memory deficit in 39.2%, executive function disorder in 9.2%, language disorder in 32.5%, behavioral disturbance in 17.5% (hypersexuality, binge eating, and frontal lobe syndrome), and psychiatric disorders in 15.8% which included mood disorders, anxiety disorder, and psychotic disorder [9]. Our patient had prominent anterograde memory deficit: he hardly remembered what he had done in the same day or the name of his doctor. He also demonstrated retrograde amnesia: he showed difficulty recalling either personal history or social events that occurred in the past decade. Amnesia and confabulations have been shown in bilateral paramedian thalamic artery infarctions in some other cases previously reported [10]. The three patients with confabulation all had bilateral involvement of the mammillothalamic tract and damage of this structure may be responsible for this unique memory disorder. The thalamus has many nuclei associated with projections to distal structures and is also proximal to other important central nervous system structures such as the midbrain and hippocampus. Some of the symptoms seen in Korsakoff syndrome may be due to involvement of median neuronal connections between the thalamus and the mammillary bodies, as evident in our patient who had retrograde amnesia and confabulations. Involvement of the dorsomedial nucleus has been previously cited as a possible cause for Korsakoff's psychosis [11]. The amnestic syndrome resulting from paramedian territory infarction is similar to the thiamine-deficient Korsakoff syndrome that destroys the medial dorsal thalamic nuclei along with the mammillary bodies [12]. Our case indicated bilateral thalamic lesions without mammillary body involvement could cause Korsakoff's syndrome. The dorsomedial nuclei, intralaminar nuclei, and mammillothalamic tract might be important structures in the pathogenesis of Korsakoff's syndrome caused by AOP infarction.

Mr. C. R. had episodes of visual and auditory hallucinations noticed during his hospital course. Organic hallucinosis refers to the occurrence of hallucinations in clear consciousness with identifiable structural etiologies. Experiential hallucinosis is a rare form of the hallucination which presents with reenactment of previous experiences in the absence of external stimuli and was reported by Penfield and Perot during artificial electrical stimulation of the temporal lobes [13]. Although the exact pathophysiology is unknown in organic hallucinosis, most cases have been described in relation to lesions in the temporal lobes, thalamus, or midbrain. Therefore, the diagnosis of organic hallucinosis seems to be more appropriate in our case. Other psychiatric disorders characterized by the presence of hallucinations were also considered. Our patient had no other psychiatric symptoms to suggest the diagnosis of schizophrenia or mood disorder.

Early recognition of AOP occlusion may help with the institution of acute stroke management and lead to favorable outcomes. The low sensitivity of CT makes AOP infarction diagnosis difficult; however it is an early screening test and may have positive results such as in our case (Figure 2(a)). MRI is the imaging modality of choice. However, one study reported normal MR (DWI) brain imaging in a comatose patient with AOP ischemic infarction [14]. The MRI (DWI) images in Percheron artery infarction can be classified as bilateral paramedian thalamic with rostral midbrain infarction (BPTRMI), bilateral paramedian thalamic without midbrain infarction (BPTWMI), bilateral paramedian and anterior thalamic with midbrain infarction (BPATMI), and bilateral paramedian and anterior thalamic without midbrain infarction [15, 16]. The most common type of infarction was BPTRMI (53%) with the most common clinical manifestations including ocular movement disorders (OMDs), aphasia or dysarthria, and behavioral amnesic impairment (BAI). In this pattern, only 25% of the patients recovered favorably at long-term follow-up, with most of the patients (75%) having an unfavorable outcome with persistent deficits. The second common type of infarction is BPTWMI, with favorable outcome in 67% of the cases, and BAI with mental status disturbance (MSD) was the most common clinical presentation in this group. The third common one, BPATMI, caused OMDs, MSD, and aphasia [16]. Our case may belong to the BPTWMI with MSD and BAI, in which there is more favorable outcome at long-term follow-up. Although AOP is rarely visualized on MR angiography, lack of visualization does not exclude its presence. Förster et al. reported 37.5% bilateral thalamic infarction patients have one or both P1 segments hypoplastic or absent, in particular 83.3% in the subgroup of patients with isolated bilateral thalamic infarction. Patients with hypoplastic/absent P1 segments were more likely to have exclusively bilateral paramedian thalamic lesions [17]. Cardioembolism is one of the most common types of etiology that occludes the AOP leading to bilateral paramedian thalamic infarctions [6]. However, only 55.6% of patients with bilateral paramedian thalamic infarctions had an embolic source. There was no significant difference with regard to frequency of embolic sources between the groups with normal or hypoplastic configuration [17]. Chuang et al. [18] reported a higher incidence of ipsilateral thalamic infarctions, especially thalamic lacunar stroke, in patients with a hypoplastic PCoA. In our case the MRA showed occluded or atretic right P1 segment, and EKG monitoring in the MICU a few days after admission detected atrial fibrillation. We presume atrial fibrillation to be the source of embolism causing P1 occlusion distal to AOP and subsequently causing AOP occlusion which resulted in his bilateral paramedian thalamic stroke. In this light, current data with our case possibly sustains the hypothesis that embolism causing AOP infarction resulting in isolated bilateral paramedian thalamic stroke might be more frequently present in patients with hypoplasia/absence or permanent/transient occlusion of the P1 segment of the PCA distal to AOP.

Successful tissue plasminogen activator (tPA) therapy has been reported in two patients, one of whom received intra-arterial thrombolysis [19] and the other received venous thrombolysis [20], respectively. As the time of onset was unknown in our patient, he was not a candidate for tPA treatment. Only 10.8% (13/120 cases) of patients with AOP were managed in the ICU after admission, but these patients had a better outcome [9]. Mortality was much higher in patients without ICU admission (13.1% versus 7.7%) [9]. Although all 13 ICU admission cases needed intubation, the literature review did not show consistent criteria for ICU admission. Our patient was admitted to ICU due to the acute altered mental status and had a favorable outcome. Long-term anticoagulant therapy is also recommended by some authors [21]. Our patient received antiplatelet treatment following standard stroke protocol initially. He was treated later with therapeutic doses of Coumadin to cover the risk of recurrent embolism from his atrial fibrillation.

## 4. Conclusion

AOP occlusion is rare. We have presented a patient with acute AOP occlusion resulting in bilateral mirror-like median thalamic infarctions involving the dorsomedial and intralaminar nuclei bilaterally. We have discussed the clinical presentations of this syndrome and the initial difficulty in diagnosis due to the presence of altered mental state only. A unique feature of this presentation included the evolution of symptoms into the Korsakoff syndrome, characterized by memory deficits and confabulation. Involvement of the mammillothalamic pathways bilaterally has been implicated in its causation. The proximity of the lesions caused by infarction in the territory of the AOP to the mammillothalamic pathways is probably the cause of the syndrome in this patient. A literature review did report a few cases with Korsakoff's syndrome in bilateral thalamic infarctions. The better outcome in relationship to management in an intensive care setting has been briefly mentioned. The importance of neuroimaging in the presence of unexplained altered mental state and cognitive difficulty to diagnose the underlying problem is important, particularly because of available therapeutic intervention in acute stroke syndromes.
